# Oral cancer awareness of undergraduate medical and dental students

**DOI:** 10.1186/1472-6920-7-44

**Published:** 2007-11-15

**Authors:** Lachlan M Carter, Graham R Ogden

**Affiliations:** 1 Specialist Registrar, Oral and Maxillofacial Surgery, Leeds Dental Institute, Clarendon Way, Leeds, LS2 9LU, UK; 2 Professor, Oral and Maxillofacial Surgery, Dundee Dental Hospital and School, Park Place, Dundee, DD1 4HN, UK

## Abstract

**Background:**

The incidence of oral cancer is increasing in the United Kingdom. Early detection of oral cancers makes them more amenable to treatment and allows the greatest chance of cure. Delay in presentation and/or referral has a significant effect on the associated morbidity and mortality. Lack of general medical practitioner and general dental practitioner oral cancer knowledge has been shown to contribute to delays in referral and treatment. The aim of this study was to investigate the oral cancer awareness of future general medical and general dental practitioners by assessing undergraduate medical and dental students' knowledge of prevention and early detection of oral cancer.

**Method:**

Questionnaires were delivered to undergraduate medical and dental students at the University of Dundee, assessing oral examination habits, delivery of advice on oral cancer risk factors, knowledge of oral cancer risk factors and clinical appearance, preferred point of referral and requests for further information.

**Results:**

Undergraduate medical students were less likely to examine patients' oral mucosa routinely and less likely to advise patients about risk factors for oral cancer. Medical students identified fewer oral cancer risk factors. In particular alcohol use was identified poorly. Medical students also identified fewer oral changes associated with oral cancer. Erythroplakia and erythroleukoplakia were identified poorly. Medical students felt less well informed regarding oral cancer. 86% and 92% of undergraduate medical and dental students respectively requested further information about oral cancer.

**Conclusion:**

This study highlights the need for improved education of undergraduate medical and dental students regarding oral cancer.

## Background

The incidence of oral cancer is increasing in the United Kingdom [1-3]. During the last decade of the 20^th ^century there was a 18% and 30% increase in oral cancer incidence in males and females respectively [1]. Despite being more prevalent in the elderly oral cancer is affecting younger patients [4]. Surgical techniques and non-surgical management of oral cancer have become more advanced in recent years but this has had little effect on 5-year survival.

Squamous cell carcinoma accounts for 95% of oral cancers and is associated with avoidable aetiological risk factors [5]. Smoking tobacco and alcohol use are the main risk factors in the United Kingdom and are associated with approximately 75% of oral cancers. Early detection of oral cancers makes them more amenable to treatment, thus reducing morbidity and allowing the greatest chance of cure [5,6]. Delay in presentation and/or referral can therefore have a significant effect on the morbidity and mortality associated with oral cancer.

Lack of public awareness has been reported in the past to be the most significant factor in delaying referral and treatment of oral cancer [7,8]. Some oral cancers may be asymptomatic [9] and thus ignorance of early signs of oral cancer may be the most important delaying factor. Lack of general medical practitioner and general dental practitioner knowledge has also been shown to contribute to delays in referral and treatment [10]. Attempts to raise oral cancer awareness of both public and health professionals have been made through initiatives like Mouth Cancer Awareness Week (MCAW) and the West of Scotland Cancer Awareness Project (WOSCAP). Although an increase in patients presenting with oral lesions and an increased referral rate by dental practitioners during the active phase of the WOSCAP campaign were reported [11], the publicity from these initiatives has had little effect on patient or referral delays [7] and public knowledge of risk factors and oral cancer signs and symptoms remains poor [12-14].

General dental practitioner oral cancer awareness is well documented. However little is known about general medical practitioner oral cancer awareness in the United Kingdom [15,16]. Similarly, whilst undergraduate dental student awareness of oral cancer and pre-malignant oral lesions has been documented [17,18], there is a paucity of information regarding undergraduate medical student oral cancer awareness in the United Kingdom.

General medical practitioners and general dental practitioners refer similar proportions of patients to maxillofacial units [10,19], and patients often consult their general medical practitioner rather than their general dental practitioner regarding oral lesions [20-22]. Thus the aim of this study was to assess the oral cancer awareness of future general medical and general dental practitioners by assessing undergraduate medical and dental students' knowledge of prevention and early detection of oral cancer.

## Method

**Figure 1:**
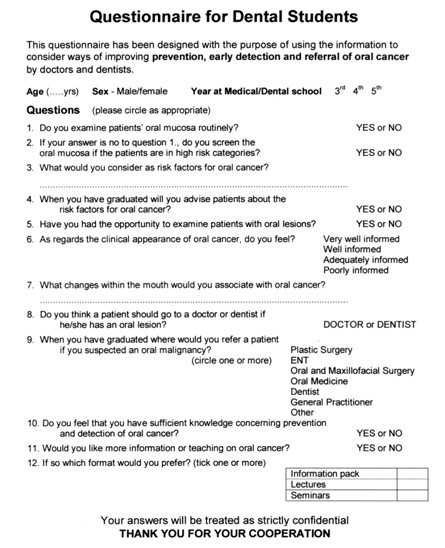
Questionnaire.

The oral cancer awareness of medical and dental students at the University of Dundee was assessed by means of a questionnaire, figure 1. The questionnaire was delivered during routine lectures to second, third, fourth and fifth year medical students and to third, fourth and fifth year dental students. These students were selected as they had received teaching on oral diseases including oral cancer. Twelve questions were asked, investigating: oral cancer screening/oral mucosal examination habits; knowledge and delivery of advice on risk factors for oral cancer; opportunity to examine patients with oral lesions; knowledge and confidence regarding appearance of oral changes associated with oral cancer; point of referral selection; and opinions on sufficiency of individual knowledge on oral cancer detection and prevention, desire for further information/training and the format of such information/training. The questionnaire required approximately ten minutes to complete. This study was undertaken during the academic year 2000/2001 and thus before the introduction of current legislation regarding ethical approval. The participating students were made aware that the data would be used for research purposes. The results were analysed using the Wilcoxon rank-sum test, and χ^2 ^test.

## Results

Questionnaires were returned by all students present during the lectures at the time of delivery. The student age and sex distribution and the number of respondents per year of course are shown in table 1.

**Table 1:** Age and sex distribution of student respondents

		Dental Students			Medical Students	
		n			n	
Total		109			255	
Fifth Year		41			41	
Fourth Year		38			55	
Third Year		30			78	
Second Year		-			81	

	Median	Inter-quartile Range	Range	Median	Inter-quartile Range	Range

Age	22	21 to 23	19 to 30	21	20 to 22	19 to 33
	Wilcoxon rank-sum test – p < 0.001					
	Male		Female	Male	Female	
Sex	44		65	99	156	
	χ^2 ^= 0.025, p > 0.1					

Significantly more dental students (99%) than medical students (28%) routinely examined patients' oral mucosa (χ^2 ^= 157.92, df = 1, p < 0.001). Of those students who did not routinely examine patients' oral mucosa, one dental student and 75 (41%) medical students would not examine the oral mucosa of high risk patients.

Question 3, "What would you consider as risk factors for oral cancer?", was asked as an open question rather than providing the answers and tick boxes. As this was an open question a wide range of responses was generated. Therefore responses in relation to diet factors and dental factors are reported as merged groups of responses. The risk factors accepted and the grouping of diet and dental factors are shown in table 2.

**Table 2:** Risk factors for Oral Cancer

Tobacco smoking	Dietary factors
Smokeless tobacco use	Diet low in iron
Betel quid chewing	Diet low in vitamin A
Alcohol consumption	Diet low in vitamin C
UV light exposure	High fat diet
Viral factors	Dental factors
Immunosuppression	Chronic irritation from jagged teeth
Chronic infection	
Occupation	'Poor dental condition' (poor oral hygiene/number of missing teeth >= 11)

**Figure 2:**
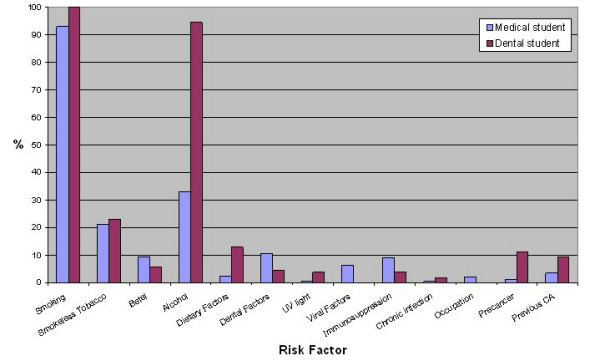
Percentage of medical and dental students identifying risk factors for oral cancer.

**Figure 3:**
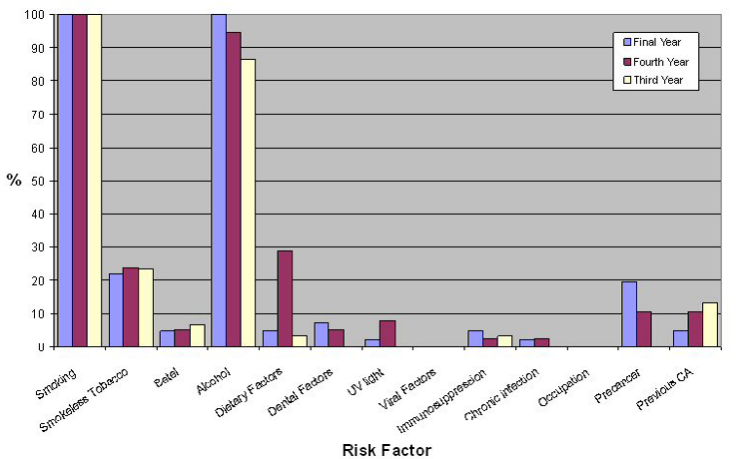
Percentage of dental students identifying risk factors for oral cancer.

**Figure 4:**
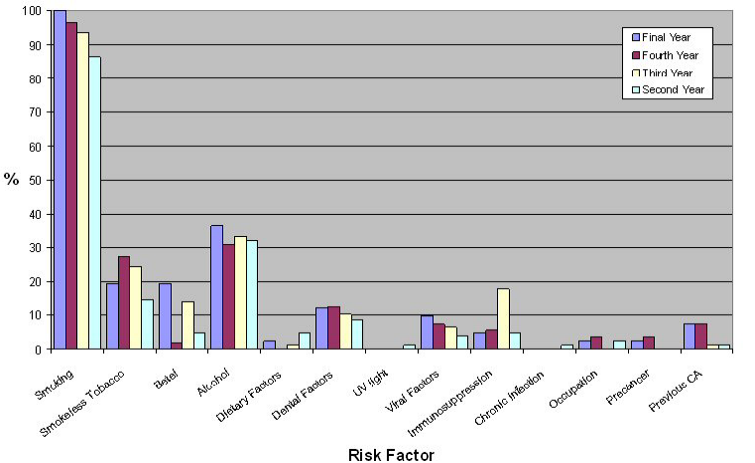
Percentage of medical students identifying risk factors for oral cancer.

Dental students identified a greater number of risk factors (median 3) than medical students (median 2, p < 0.001). The distribution of risk factors identified is shown in figures 2, 3 and 4. Significantly fewer medical students identified smoking (medical 93% and dental 100%, χ^2 ^= 60.15, df = 1, p < 0.001) and alcohol (medical 33% and dental 94%, χ^2 ^= 60.15, df = 1, p < 0.001) as risk factors. Significantly more dental students reported that they would advise patients regarding oral cancer risk factors after graduation (medical 67% and dental 93%, χ^2 ^= 27.87, df = 1, p < 0.001).

Significantly more final year dental students than medical students reported having had the opportunity to examine patients with oral lesions (dental 88% and medical 61%, χ^2 ^= 6.401, df = 1, p = 0.0122) and significantly more dental students felt very well or well informed regarding the clinical appearance of oral cancer (χ^2 ^= 68.32, df = 1, p < 0.001).

**Figure 5:**
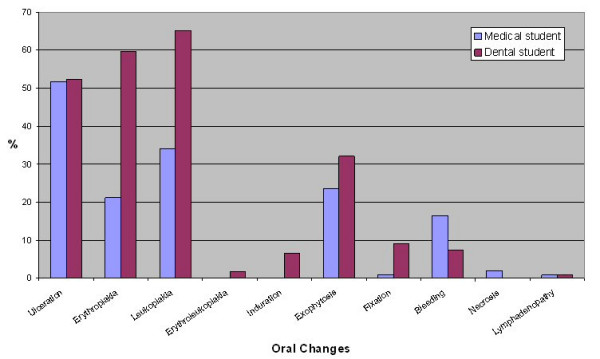
Percentage of medical and dental students identifying oral changes associated with oral cancer.

**Figure 6:**
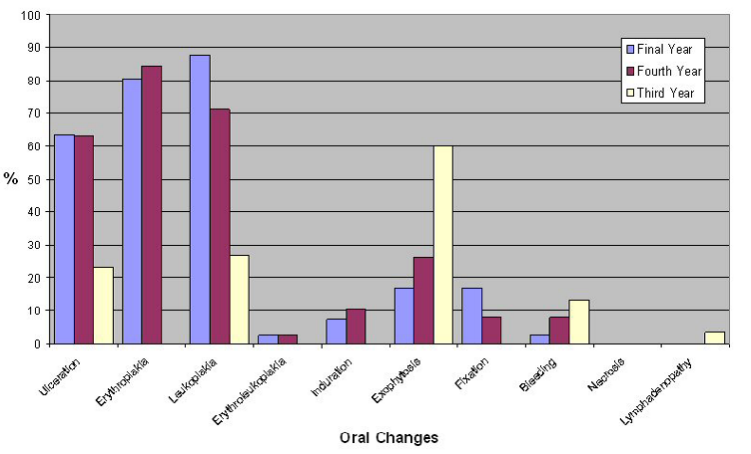
Percentage of dental students identifying oral changes associated with oral cancer.

**Figure 7:**
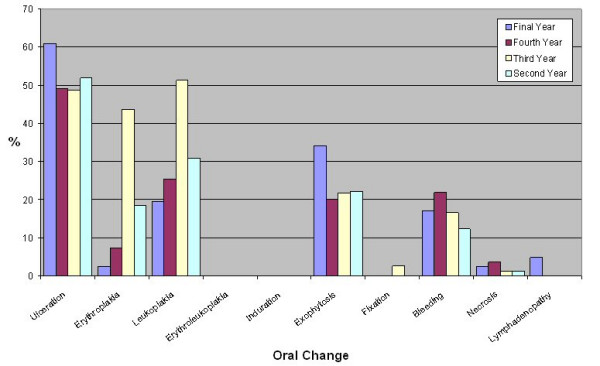
Percentage of medical students identifying oral changes associated with oral cancer.

Question 7, "What changes within the mouth would you associate with oral cancer?", was again asked as an open question rather than providing the answers and tick boxes. Again as this was an open question a wide range of responses was generated. Therefore responses in relation to exophytosis are reported as a merged group of responses. The oral changes accepted and the exophytosis grouping are shown in table 3. Dental students identified a greater number of oral changes (median 3) than medical students (median 1, p < 0.001). The distribution of oral changes identified is shown in figures 5, 6 and 7. Significantly more dental students identified erythroplakia (χ^2 ^= 16.96, df = 1, p < 0.001) and leukoplakia (χ^2 ^= 16.96, df = 1, p < 0.01) as oral changes associated with oral cancer. Other oral changes were identified poorly by both medical and dental students.

**Table 3:** Oral changes associated with Oral Cancer

Ulceration	Exophytosis
Erythroplakia	Mass
Leukoplakia	Lump
Erythroleukoplakia	Growth
Induration	Necrosis
Fixation	Lymphadenopathy
Bleeding	

**Figure 8:**
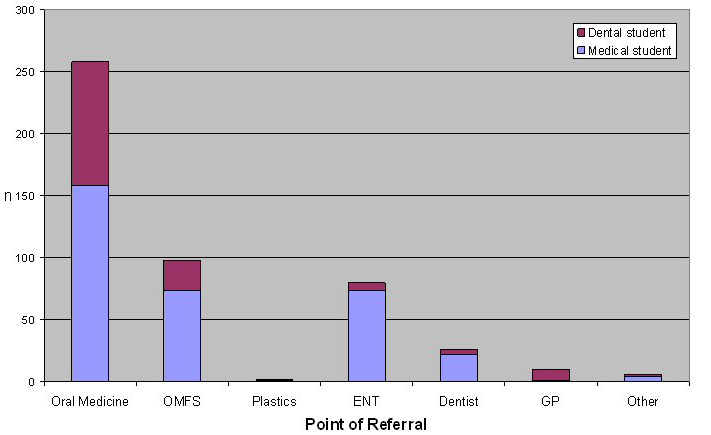
Point of referral selected by medical and dental students.

The majority of medical and dental students selected Oral Medicine and Oral and Maxillofacial Surgery as the point of referral for a patient with an oral cancer, see figure 8.

Significantly more dental students felt that they had sufficient knowledge regarding prevention and early detection of oral cancer (χ^2 ^= 63.52, df = 1, p < 0.001), however 34% and 93% of final year dental and medical students respectively felt that they did not have sufficient knowledge regarding prevention and early detection of oral cancer.

Approximately ninety percent of both medical and dental students requested further information on prevention and early detection of oral cancer with an information pack being the most popular form of further information.

## Discussion

Dental students were significantly older than medical students. This can be explained by the inclusion of second year students in the medical students group. The groups were similar in distribution of males and females.

Not surprisingly significantly more dental students routinely examined patients' oral mucosa. Medical students may examine patients' oral mucosa in relation to the context of the consultation, for example presentation with an oral problem. General medical practitioners are more likely to see patients at higher risk of oral cancer [21,22]. Medical students are also more likely to see patients at higher risk of oral cancer than their dental counterparts and yet 42% of medical students would not examine the oral mucosa of high risk patients whereas only 1 dental student would not.

Smoking tobacco as a risk factor was identified well by both medical and dental students however significantly more dental students identified this risk factor. Significantly more dental students (94%) than medical students (33%) identified alcohol as a risk factor. This is consistent with previous literature regarding general medical practitioners [15,16,23,24]. Thus the role of alcohol as a risk factor for oral cancer has to be emphasised in future teaching of undergraduate medical students. Knowledge of other risk factors was poor in both medical and dental students. There was a trend toward increased risk factor identification from second to fifth year medical students and from third to fifth year dental students. Comparison of risk factor knowledge amongst students at different years of training can be difficult to interpret as curricular factors, public awareness campaigns and changes in faculty can contribute to changes in risk factor knowledge.

Significantly more dental students reported that they would advise patients regarding oral cancer risk factors after graduation. This is similar to previous studies regarding smoking cessation advice by dental students [25] and may be related to greater risk factor knowledge, but is not consistent with studies regarding the actual practice of oral cancer risk factor counselling by general dental practitioners [23,26].

Oral changes associated with oral cancer were identified less well than risk factors by both medical and dental students. Dental students identified more risk factors than medical students. Significantly more dental students identified erythroplakia and leukoplakia as oral changes. Despite the malignant potential of these lesions, erythroplakia was not frequently identified by medical students and erythroleukoplakia was poorly recorded by both medical and dental students. Histopathologically, it has been documented that in homogenous erythroplakia 51% showed invasive carcinoma [27] and the malignant transformation rate of erythroplakia and erythroleukoplakia can be at least 50% [28]. Leukoplakia has less malignant potential than erythroplakia, however non-homogenous, speckled and nodular types of leukoplakia can have similar rates of malignant transformation to erythroplakia [29,30]. In a recent paper using closed questions approximately 1 in 3 people were aware of white patches (leukoplakia) and 1 in 4 people were aware of red patches (erythroplakia) as early signs of oral cancer [13]. In our study the question was open – and 34% and 21% of medical students respectively knew that white patches (leukoplakia) and red patches (erythroplakia) were early signs of mouth cancer. Although this level of knowledge is better than that of general medical practitioners [16,31] it is similar to that of the general population. The significance of leukoplakia, erythroplakia and erythroleukoplakia needs to be emphasised in future teaching of both medical and dental students. Poor knowledge of oral changes may be related to the level of teaching received, as approximately 40% of medical students reported not having had the opportunity to examine patients with oral lesions, and significantly more medical students felt less well informed regarding the clinical appearance of oral cancer.

Oral Medicine and Oral and Maxillofacial surgery were the most commonly selected proposed points of referral for patients with a suspected oral cancer. The availability of Dundee Dental Hospital within Tayside may explain why a greater proportion of students selected Oral Medicine. This may not be generalisable to other regions without a dental hospital. There is also the possibility that students were steered towards referring to Oral Medicine and Oral and Maxillofacial Surgery units by virtue of the word 'oral' appearing in the title. In retrospect, bias may have been reduced if this question was left open to the respondents rather than presented as a closed question.

93% of final year medical students felt they did not have sufficient knowledge regarding prevention and early detection of oral cancer. This is consistent with previous studies of general medical practitioners where confidence about oral cancer knowledge was attributed to a lack of training [23]. Medical students have less risk factor knowledge, less knowledge of oral changes associated with oral cancer and are less likely to examine the oral mucosa of patients, including those at high risk of oral cancer, than dental students. However patients often present to their general medical practitioner regarding oral lesions and general medical practitioners are more likely to see patients at higher risk of oral cancer [20-22].

In comparison with previous studies, medical students had similar oral cancer knowledge to general medical practitioners, showing no improvement in the next generation of doctors [16,31]. Oral cancer awareness of medical students could be improved by maximising the opportunities for teaching regarding oral health and disease within already crowded medical curricula. Unfortunately oral health has traditionally received little emphasis in medical curricula in the past [32]. Opportunities for clinical teaching regarding oral cancer for medical undergraduates may present during clinical attachments in Oral and Maxillofacial Surgery, Otorhinolaryngology, Plastic Surgery or Clinical Oncology. A collaborative approach from these specialties ensuring the opportunity to take oral health histories and examine patients with oral lesions before graduation should be undertaken. Interprofessional collaboration with dental surgeons has also been suggested for both teaching and assessment [33]. The medical undergraduate curriculum at the University of Dundee now benefits from the inclusion of teaching on oral diseases by Oral and Maxillofacial Surgeons, as part of the gastrointestinal system teaching. In addition to formal lectures on oral and dental health this has allowed development of clinical skills sessions in identification of oral mucosal lesions and dental disease, taking oral health histories and examination of the face, jaws, oral cavity and neck (including cervical lymphadenopathy). These teaching sessions have been favourably received by the students [34]. Therefore a more proactive approach from oral and maxillofacial surgeons and oral physicians toward teaching medical undergraduates should be undertaken. Special study modules or electives in oral health and disease may improve oral health awareness of both undergraduates and faculty from non oral health related specialties [35], although voluntary modules may attract those students with an existing interest in oral health and disease. 75% of oral cancers are associated with smoking tobacco and alcohol use. These risk factors are common to many aero-digestive tract cancers as well as cardiovascular and liver disease and therefore oral cancer should be included as part of teaching regarding tobacco and alcohol related health issues.

## Conclusion

As the incidence of oral cancer continues to rise in the United Kingdom, the role that general medical (and dental) practitioners may play in prevention and detection of oral cancer assumes ever more importance. This study showed persistence of a poor level of awareness regarding oral cancer in the next generation of general medical practitioners and therefore highlights the need to improve the education of undergraduate medical and dental students regarding prevention and early detection of oral cancer.

## Competing interests

The author(s) declare that they have no competing interests.

## Authors' contributions

LC delivered the questionnaire to the undergraduate medical students at Dundee University Medical School, performed the data analysis and drafted the manuscript. GO conceived the study and delivered the questionnaire to the undergraduate dental students at Dundee University Dental School. Both authors read and approved the final manuscript.

## Pre-publication history

The pre-publication history for this paper can be accessed here:


